# Can Large Language Models Replicate Systematic Review Outcome Classifications in Medical Education? A Pilot Study Using Kirkpatrick Levels

**DOI:** 10.1007/s40670-026-02639-1

**Published:** 2026-01-16

**Authors:** Giuliano Romano, Emilio Romano, Michelle Rau

**Affiliations:** 1 Oakland University William Beaumont School of Medicine, Rochester, MI United States of America; 2 University of Michigan College of Medicine, Ann Arbor, MI United States of America

**Keywords:** Generative AI, ChatGPT, Artificial intelligence in medical education, Systematic reviews, Kirkpatrick framework

## Abstract

Systematic reviews in medical education often classify outcomes using the Kirkpatrick framework, but manual coding is time-consuming and subjective. We conducted a proof-of-concept study testing ChatGPT (GPT-5, August 2025 release) on 32 full-text articles from a published systematic review of sepsis education. Agreement with human-coded outcomes was modest: 50% percent agreement, unweighted κ = 0.170 (95% CI 0.000–0.458), weighted κ = 0.351 (95% CI 0.074–0.629). Most disagreements were between adjacent levels.

**Supplementary Information:**

The online version contains supplementary material available at 10.1007/s40670-026-02639-1.

## Background

Systematic reviews are widely used in medical education to synthesize evidence on educational interventions and guide education practices. Within health professions education, the four-level Kirkpatrick model is a commonly used framework for classifying outcomes in systematic reviews. Numerous reviews have organized their findings according to Kirkpatrick levels when evaluating interventions across multiple domains, including interprofessional simulation, crisis management simulation, near-peer surgical teaching, virtual reality training, and skills-focused simulation reviews [1–5]. This model provides a simple, structured approach to evaluating the impact of educational activities at multiple levels of effect: Level 1 (Reaction), learner satisfaction or perceived value of the intervention; Level 2 (Learning), change in knowledge, skills, or attitudes; Level 3 (Behavior), translation of learning into practice or behavior change; and Level 4 (Results), impact on organizational outcomes or patient care. While widely used, the Kirkpatrick model has been critiqued for limitations involving complex educational interventions, with calls for more nuanced evaluative approaches [6].

While systematic reviews are the cornerstone of evidence-based practices, conducting systematic reviews remains a labor and time intensive endeavor. Most systematic reviews take 1–2 years to complete [7, 8]. Contributing factors to the time burden of conducting systematic reviews include intensive steps such as study selection, data extraction, and critical appraisal, which largely remain manual processes [9]. While automation and machine learning tools are already being used to assist in screening, data extraction, and summarization in systematic reviews, there is limited data supporting their use for other parts of the review process including full text data extraction [10–12].

Large language models (LLMs) such as ChatGPT are increasingly being used and tested in medical education for applications including academic assistance, scenario simulation and curriculum development [13, 14]. LLMs’ ability to process large volumes of text quickly and recognize patterns may make them a valuable tool for data extraction and outcome classification, thereby reducing workload and time needed to complete systematic reviews. However, to our knowledge no study has evaluated their potential for classifying outcomes in systematic reviews. Our investigation aimed to assess ChatGPT’s ability to perform data extraction and classification at the full article level using the Kirkpatrick model as framework.

## Activity

A proof-of-concept study was conducted using secondary analysis of published articles. The study’s goals were to assess feasibility of using ChatGPT to extract Kirkpatrick outcome classification and compare ChatGPT’s Kirkpatrick outcome classification to human-coded outcomes from a published systematic review. The primary outcome was percent agreement between ChatGPT’s Kirkpatrick classification and the review authors’ highest reported classification for each study.

A published systematic review article was used to serve as a standard of comparison because it provided an established, peer-reviewed benchmark of Kirkpatrick outcome classifications while ensuring transparency and reproducibility. Criteria for selection of the systematic review included evaluating an educational intervention using the Kirkpatrick framework, sample size ≥ 20 articles, structured table reporting of outcomes and open access. The systematic review by Choy et al. was found to meet these criteria. All 32 studies reported in the review were included in analysis. In the reference review, study quality was appraised independently by two reviewers with consensus resolution. For our LLM, ChatGPT (GPT-5, August 2025 release) was utilized via the OpenAl web platform. Parameters such as temperature and maximum token length were determined by the platform defaults and could not be manually adjusted.

PDFs of all studies in the review were obtained via institutional access or open access. A structured JSON prompt (see Online Resource 1) guided ChatGPT through the following steps: (1) extracting and segmenting relevant article text; (2) dividing long sections into manageable chunks when needed; (3) assigning a Kirkpatrick level to each chunk with justification; and (4) aggregating classifications across chunks to identify the highest defensible Kirkpatrick level at the article level. Ideas for prompt structure and wording were first generated using ChatGPT to explore strategies for clearly instructing the model to identify and classify outcomes according to the Kirkpatrick framework. The study team then refined the prompt to ensure alignment with accepted definitions of Kirkpatrick levels and output included all information prompted. In this study, the highest defensible Kirkpatrick level was defined as the maximum level supported by explicit outcome statements within the article. ChatGPT was instructed to provide direct evidence and a brief rationale to justify its classification. To ensure consistent interpretation, the prompt explicitly included definitions of each Kirkpatrick level, so that ChatGPT classified outcomes according to a standardized rubric rather than relying on implicit knowledge. To ensure reproducibility, each data extraction was initiated in a single new thread with explicit instructions to ignore all prior context, so that only the study prompt and article text were considered. Before applying the prompt to the full dataset, it was tested on a single article to evaluate clarity, output structure, and alignment with Kirkpatrick level definitions. The prompt was entered, and each article PDF was uploaded sequentially, with each JSON output logged before uploading the next. Outputs were transcribed to Excel for comparison to outcomes reported in Table II from Choy et al. [15].

### Results & Discussion

A total of 32 studies were included in the final analysis. In the reference article, Choy et al. assigned most articles to Kirkpatrick Level 2 (*n* = 17, 53%), followed by Level 3 (*n* = 9, 28%) and Level 4 (*n* = 6, 19%). No studies were classified at Level 1. ChatGPT assigned most articles to Kirkpatrick Level 2 (*n* = 19, 59%), followed by Level 4 (*n* = 8, 25%) and Level 3 (*n* = 4, 13%), with one study classified at Level 1.

To illustrate how ChatGPT derived its classifications, Table 1 presents representative examples of studies along with ChatGPT’s extracted outcome statements and explanations supporting the assigned Kirkpatrick levels.

**Table 1:** Examples of ChatGPT outcome classification Output, including extracted evidence and rationale for assigned Kirkpatrick levels

Study	Kirkpatrick Level Assigned	Evidence Extracted by ChatGPT	Rationale Provided by ChatGPT
Adhikari_2021	2	“chunk_id”: “Results”, “quoted_sentence”: “There was a 26.1% increase in mean confidence score post-intervention (86.9 (SD 19.2) vs 109.6 (SD 22.3) (P < 0.001)).” “chunk_id”: “Results” “quoted _sentence”: “Similarly, there was a 23.4% decrease in anxiety (77.4 (SD = 12.5) vs 59.3 (SD = 15.9) (P < 0.001)).” “chunk_id”: “Conclusions” “quoted _sentence”: “Evaluation of participant’s perception showed that the IVR game has a positive impact on knowledge, decision-making skills and confidence outcome.”	“rationale”: “Across results, discussion, and conclusions, outcomes focus on changes in confidence, anxiety, and self-reported perceptions of knowledge and decision-making. These reflect modifications of attitudes/perceptions rather than objective tests of knowledge/skills, behavior in practice, or patient/organizational outcomes. Therefore, the highest defensible level is Kirkpatrick Level 2a.”
Bolger_2017	4	“chunk id”: “Results”. “quoted_sentence”: “Mean variable scores: Q1 = 5.58, Q2 = 5.06, Q3 = 5.76, Q4 = 6.20.” “chunk id”: “Results” “quoted _sentence”: “The result would indicate there was no statistically significant increase from quarter 1 to quarter 4.” “chunk_id”: “Discussion”, “quoted sentence”: “The findings of the audit show that, although there was a slight increase in the mean average from quarter 1 through to quarter 4, there was no statistically significant increase in maternity staff ‘s compliance with the Trust maternal sepsis guideline, as documented in the maternity notes.” “chunk id”: “Conclusion _Implications”, “quoted _sentence”: “The audit indicates the maternal sepsis training package had little significant impact on compliance.”	“rationale”: “The outcomes evaluated were compliance rates with the Trust’s maternal sepsis guidelines, representing changes in organizational practice attributable to training. Although no statistically significant improvement was found, the study directly measured system-level practice outcomes. This aligns with Kirkpatrick Level 4a (Change in organizational practice).”

ChatGPT matched the Choy et al. [15] classifications in 16 of 32 studies (50%). The unweighted Cohen’s κ was 0.170 (95% CI, 0.000–0.458). The weighted κ was 0.351 (95% CI, 0.074–0.629).

**Fig. 1:**
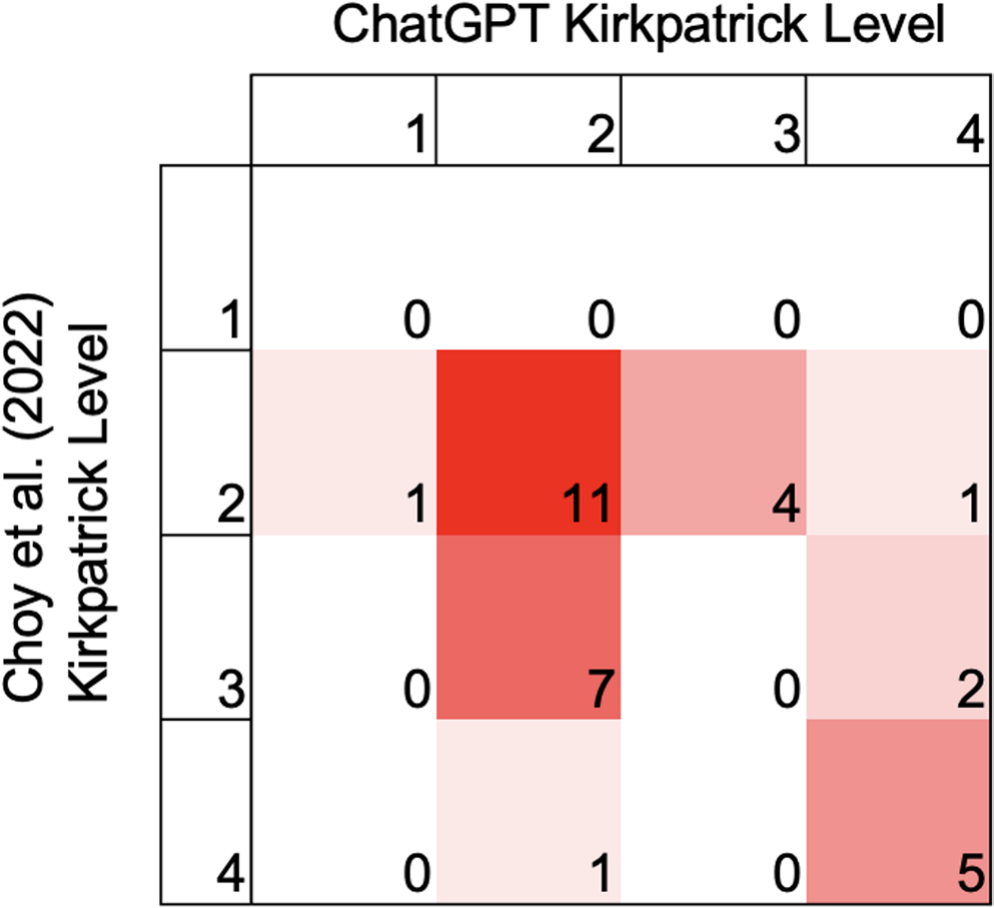
Agreement between ChatGPT and Choy et al. (2022) on Kirkpatrick Classification

The distribution of classifications assigned by ChatGPT compared to Choy et al., [15] is illustrated in a confusion matrix heatmap (Fig. 1). The heat map shows most agreement between ChatGPT and the reference article occurred in the Level 2 classification. Disagreements were most common between the Level 2 and Level 3 classifications. Large misclassifications, such as between Levels 4 and Level 1 or 2 classification were rare.

Figure 1 Heatmap comparing Kirkpatrick levels assigned by ChatGPT and Choy et al. Numbers indicate the count of studies in each classification cell. Shading reflects frequency, with darker red indicating higher counts.

The purpose of this study was to evaluate the feasibility of using ChatGPT to extract Kirkpatrick outcome classification and compare ChatGPT’s Kirkpatrick outcome classification to human-coded outcomes from a published systematic review. This is the first pilot to test LLMs on outcome classification at full-text level in medical education. In terms of feasibility, ChatGPT was able to classify full-text educational studies into Kirkpatrick levels and provide evidence and rationale for the level selected. However, agreement was modest with the published systematic review at 50%. This indicates while agreement was not perfect, LLMs can engage with structured outcome frameworks.

Using Landis and Koch’s [16] interpretation of kappa values, the unweighted κ of 0.17 corresponds to slight agreement between reviewers, while the weighted κ of 0.35 corresponds to fair agreement. Most disagreements between the reviewers occurred between adjacent Kirkpatrick levels, as evidenced by the weighted κ being greater than the unweighted κ. It is important to consider this in comparison to human reviewers, who also may disagree with each other when classifying articles that fall into a gray area in between two adjacent levels.

This proof-of-concept study shows that while ChatGPT can extract and classify data at the full text level, it is not highly reliable when compared to human reviewers. Even so, disagreements were often close in levels, indicating that while ChatGPT is not at the level where it can replace human reviewers, it still has the potential to be used as an aid for data extraction when conducting systematic reviews. For example, it could be used as a first past screener that flags ambiguous cases for human review, saving time and improving workflow efficiency. However, caution should be used, as using LLMs as a first pass screener could introduce confirmation bias to studies. If developed further, LLMs such as ChatGPT can serve as a validated tool for data extraction and classification, allowing for semi-automated evidence synthesis and greater reproducibility by enforcing consistent decision rules.

As a pilot proof-of-concept study, there are many limitations to this study. Although peer reviewed, using a published systematic review as the gold standard for comparison is not a perfect standard as the review and classification process is subjective among the authors. Therefore, our results reflect agreement with one human dataset rather than an absolute objective standard. Furthermore, our analysis compared only the highest Kirkpatrick level per study. While consistent with common practice, this may overlook agreement at lower levels. Additionally, using a single systematic review dataset limits the generalizability of the study.

Future research can expand on this proof-of-concept by applying the method across multiple systematic reviews from different topics to improve generalizability. Additionally, future work should systematically develop and test prompts across varied content areas to ensure reliability and generalizability, as intentional prompt engineering may further improve agreement between ChatGPT classifications and human reviewers. Lastly, future studies can explore how the LLM-human inter-rater reliability compares to human-human inter-rater reliability for better context on how LLMs perform compared to traditional human raters in systematic review coding.

This study demonstrates that large language models such as ChatGPT are capable of extracting and classifying outcomes from full-text educational studies using the Kirkpatrick framework. While agreement with a published systematic review was modest, disagreements most often occurred between adjacent levels. These findings support the potential assistive role of LLMs in conducting systematic reviews, rather than replacement for human reviewers. With further refinement, LLMs may help reduce the obstacles individuals face when conducting systematic reviews.

## Supplementary Information

Below is the link to the electronic supplementary material.

Supplementary Material 1 (PDF 48.9 KB)

## Data Availability

The datasets generated and analyzed during the current study are available from the corresponding author on reasonable request.

## Declarations

This study was a secondary analysis of previously published articles and did not involve human participants, their data, or biological material.
