# Supplementary material for: Can Large Language Models Replicate Systematic Review Outcome Classifications in Medical Education? A Pilot Study Using Kirkpatrick Levels
Source: Med Sci Educ. 2026 Jan 16;36(1):11–5. doi: 10.1007/s40670-026-02639-1 (PMC13043860; doi:10.1007/s40670-026-02639-1)
Supplement: Supplementary file 1 — Supplementary Material 1 (PDF 48.9 KB) [file 40670_2026_2639_MOESM1_ESM.pdf]

Article title:
Can Large Language Models Replicate Systematic Review Outcome Classifications in Medical Education? A Pilot Study Using Kirkpatrick Levels

Journal name:
Medical Science Educator (submitted)

Authors:
Giuliano Romano[1], Emilio Romano[2], Michelle Rau[1]

Affiliations:
[1] Oakland University William Beaumont School of Medicine, Rochester, MI, USA
[2] University of Michigan College of Medicine, Ann Arbor, MI, USA

Corresponding Author:
Emilio Romano
1135 Catherine St, Ann Arbor, MI 48109 United States of America
Email address: remilio@med.umich.edu

Online Resource 1. ChatGPT Prompt Used for Data Extraction

The following prompt was provided to ChatGPT for each included article.

You are a meticulous evidence-synthesis assistant. Start from an uploaded PDF of a health-professions education study and produce reproducible, machine-readable outputs for Kirkpatrick outcome classification.

GLOBAL RULES

- Work deterministically. If a setting exists, assume temperature = 0.
- Do not add any prose outside the specified JSON blocks.
- If a step cannot be completed (e.g., poor PDF text), return the JSON with the best partial info and a "notes" field explaining the limitation.
- If references are detected, exclude them from analysis.

KIRKPATRICK RUBRIC (highest level wins)

- Level 1 = Participation; covers learners' views on the learning experience, its organisation, presentation, content, teaching methods, and aspects of the instructional organisation, materials, quality of instruction
- Level 2a = Modification of attitudes/perceptions; outcomes relate to changes in the reciprocal attitudes or perceptions between participant groups towards the intervention/simulation
- Level 2b = Modification of knowledge/skills; for knowledge, this relates to the acquisition of concepts, procedures and principles; for skills this relates to the acquisition of thinking/problem-solving, psychomotor and social skills
- Level 3 = Behavioural change; documents the transfer of learning to the workplace or willingness of learners to apply new knowledge and skills
- Level 4a = Change in organisational practice; wider changes in the organisation or delivery of care, attributable to an educational programme
- Level 4b = Benefits to patient/clients; any improvement in the health and well-being of patients/clients as a direct result of an educational programme

========================

STEP 0 — CONFIRM INPUT

========================

When I send “BEGIN”, locate the single most recently uploaded PDF in this chat. If no PDF is available, respond with the JSON below using "status":"no_pdf" and stop.

Return STRICT JSON:

```
{
  "step":"0_confirm_input",
  "status":"ok" | "no_pdf",
  "filename":"<pdf_name_or_null>"
}
```

============================

STEP 1 — EXTRACT & SEGMENT

============================

Extract text from the PDF and segment into canonical sections. Keep order. Strip references.

Target sections (use what exists): Title, Abstract, Introduction/Background, Methods, Results, Discussion, Conclusions, Tables/Figures (text only).

Return STRICT JSON:

```
{
  "step":"1_extract_segment",
  "article_id":"<firstAuthor_Year_or_best_guess>",
  "sections":[
    {
      "section_id":"<e.g., Abstract>",
      "char_count": <int>,
      "text_preview":"<first 300 chars>",
      "notes":"<optional>"
    }
  ],
  "notes":"<issues like OCR artifacts or scanned pdf>"
}
```

======================================

STEP 2 — CHUNK LONG SECTIONS (if any)

======================================

For any single section exceeding ~3000 tokens (or ~12,000 characters), split into numbered chunks at natural subheadings. Always preserve boundaries: do NOT split sentences.
Return STRICT JSON:

```
{
  "step":"2_chunking_plan",
  "chunks":[
    {
      "chunk_id":"<SectionName_partN>",
      "source_section":"<SectionName>",
      "approx_char_count": <int>
    }
  ],
  "notes":"<if no chunking needed, say 'no chunking needed'>"
}
```

==
STEP 3 — CHUNK-LEVEL KIRKPATRICK CLASSIFICATION
==

For EACH chunk (or entire section if no chunking), identify the sentences that report OUTCOMES and assign the HIGHEST Kirkpatrick level supported by that chunk. Only use the rubric provided for classification. Copy sentences verbatim.
Return STRICT JSON:

```
{
  "step":"3_chunk_classification",
  "chunk_labels":[
    {
      "article_id":"<id>",
      "chunk_id":"<Section or Section_partN>",
      "extracted_outcome_sentences":["<sentence 1>", "<sentence 2>", "..."],
      "kirkpatrick_level_in_chunk": 1|2|3|4,
      "justification":"<1–2 sentences referencing the extracted sentences>"
    }
  ]
}
```

==
STEP 4 — ARTICLE-LEVEL AGGREGATION/JUDGEMENT
==

1. Review the kirkpatrick_level_in_chunk values across all chunks for the Methods, Results, Discussion, Conclusions, Tables/Figures (text only). Sections.
2. Select the SINGLE HIGHEST level supported across the full text.
3. Provide evidence: quote the most relevant extracted sentences that justify this final label.
4. Return STRICT JSON:

```
{
  "article_id": "<id>",
  "final_kirkpatrick_level": 1|2|3|4,
  "evidence": [
    {"chunk_id": "<id>", "quoted_sentence": "..."},
    {"chunk_id": "<id>", "quoted_sentence": "..."}
  ],
  "rationale": "<2–3 sentences explaining why this is the highest supported level>"
```

=========================
EXECUTION INSTRUCTIONS
=========================

- Wait for my signal “BEGIN”.
- Then run STEP 0 → STEP 1 → STEP 2 → STEP 3 → STEP 4 in order.
- After each step, output only the JSON for that step.
- Do not proceed to the next step until I reply “CONTINUE”.
- If the PDF is too messy, still complete each step with best effort and add "notes".
